# Comparing Frailty and Traditional Risk Models in Predicting 6-Month Mortality After Hip Fracture in Older Adults: A Retrospective Study from a Single Center

**DOI:** 10.3390/jcm15145625

**Published:** 2026-07-17

**Authors:** Merve Güner, Eda Ural Karaman, Yavuz Şahbat

**Affiliations:** 1 Division of Geriatric Medicine, Erzurum City Hospital, Ministry of Health, Erzurum 25240, Türkiye; 2 Department of Internal Medicine, Faculty of Medicine, Istinye University, Istanbul 34010, Türkiye; 3 Department of Internal Medicine, Erzurum City Hospital, Ministry of Health, Erzurum 25240, Türkiye; eda.ural@atauni.edu.tr; 4 Department of Orthopedics and Traumatology, Erzurum City Hospital, Ministry of Health, Erzurum 25240, Türkiye; yavuz.sahbat@isu.edu.tr; 5 Department of Orthopedics and Traumatology, Istinye University Liv Bahçeşehir Hospital, Istanbul 34517, Türkiye

**Keywords:** hip fracture, mortality prediction, frailty assessment, risk stratification, geriatric surgery, survival analysis, perioperative risk assessment

## Abstract

**Background**: Mortality risk in patients with hip fractures is influenced by multiple factors, including age, pre-existing medical conditions, and frailty. This study aims to compare the predictive ability of 6-month mortality between traditional risk models—the American Society of Anesthesiologists (ASA) classification, the Hip Fracture Estimator of Mortality of Amsterdam (HEMA), and the Nottingham Hip Fracture Score (NHFS)—and frailty assessment. **Methods**: This retrospective study included patients who were admitted to the Orthopedics and Traumatology Clinic with intracapsular femoral neck fractures treated with hemiarthroplasty and with extracapsular intertrochanteric fractures treated with intramedullary nailing between 1 January 2023 and 31 December 2023. Demographic and clinical data were retrieved from medical records, and survival status at 6 months post-admission was determined using the Turkish Death Registry System. The frailty status was determined retrospectively from electronic medical records using the Clinical Frailty Scale (CFS) by a trained specialist. **Results**: A total of 164 patients were analyzed, with a mean age of 78.2 ± 8.9 years, and 64.6% (*n* = 106) were female. The 6-month mortality rate was 31.1% (*n* = 51). Frailty, as measured by CFS, was significantly associated with increased mortality risk [hazard ratio: 4.465, 95% confidence interval (CI): 2.588–7.703, *p* < 0.001]. Receiver Operating Characteristic (ROC) analysis demonstrated that all risk models were predictive of 6-month mortality, with CFS exhibiting the highest area under the curve (AUC) (CFS: AUC = 0.879 (95% CI: 0.824–0.934, *p* < 0.001). **Conclusions**: In this cohort, several perioperative risk scores were associated with mortality; however, frailty measured by the CFS provided stronger discrimination for medium-term prognosis after hip fracture surgery. Incorporating frailty assessment alongside perioperative risk evaluation may improve risk stratification and support orthogeriatric care planning and postoperative management in this high-risk population.

## 1. Introduction

Hip fractures are a significant health issue, especially among older adults, as they can severely impact mobility, independence, and overall well-being [1]. These fractures often result in extended hospital stays and carry a high risk of complications, such as infections, cardiovascular events, a loss of physical function, and high health expenditures [2,3]. Hip fractures are associated with high mortality rates, approximately 7.1% within the first month and 30% within the first year post-fracture [4]. Mortality risk in this population is influenced by various factors, including age, pre-existing medical conditions, poor nutritional status, cognitive impairment, and frailty [5,6]. A patient’s functional level before the fracture, their ability to regain mobility post-surgery, and the quality of surgical and postoperative care also play crucial roles in survival [7,8].

Mortality rates following hip fracture surgery are notably high, and preoperative risk assessment remains a complex task. Risk models, including the American Society of Anesthesiologists (ASA) classification, the Hip Fracture Estimator of Mortality of Amsterdam (HEMA), and the Nottingham Hip Fracture Scale (NHFS), could help identify patients needing intensive perioperative care, provide prognostic insights, and enable clinicians to anticipate patient outcomes and tailor interventions [9,10,11,12]. Risk prediction models aim to stratify patients based on their probability of survival following a hip fracture. However, predicting individual patient mortality at the time of hospital admission remains challenging. Increasingly, frailty has been recognized as a critical factor for assessing risk, offering a comprehensive evaluation of patient complexity [13,14].

Frailty is a clinical syndrome characterized by diminished physiological reserves, impaired cognition, and a higher vulnerability to adverse health outcomes [15]. While related to chronological age and comorbidities, frailty is a distinct entity requiring careful assessment. Although no universally accepted definition exists, two primary frameworks for frailty assessment are widely used: the deficit accumulation index and the physical frailty [16,17]. The Clinical Frailty Scale (CFS), developed by Rockwood and colleagues as part of the Canadian Study of Health and Aging, is a widely used tool in clinical practice [18]. This scale summarizes frailty and is based on clinicians’ judgment, integrating evaluations of patients’ overall health status [18].

The CFS is widely used to assess frailty in older adults hospitalized with acute illness, has been associated with inpatient and post-discharge outcomes, and can support the allocation of orthogeriatric resources within hospitals [19,20]. Although several studies have examined frailty in relation to mortality and postoperative complications after hip fracture, results are not fully consistent [21]. Moreover, evidence remains limited regarding how frailty assessment compares with, or complements, commonly used perioperative risk tools in predicting medium-term mortality after hip fracture surgery. Therefore, the aim of this study was to evaluate the prognostic performance of CFS for predicting 6-month mortality following hip fracture surgery and to compare its discriminative performance with that of traditional perioperative risk models.

## 2. Materials and Methods

### 2.1. Study Population

Two hundred and ten patients who were admitted to the Orthopedics and Traumatology clinic and underwent surgery for hip fractures between 1 January 2023 and 31 December 2023 were retrospectively identified in the hospital information system. Patients younger than 60 years (32 patients) were excluded from the study. Four patients with hip fractures resulting from major trauma, such as a car accident or a fall from a higher level, were also excluded. The patients who had missing data in their files for the study (14 patients) were not included in the study. Hip fractures were classified as intracapsular and extracapsular fractures. Intracapsular fractures comprised femoral neck fractures (subcapital, transcervical, and basicervical), whereas extracapsular fractures included intertrochanteric and subtrochanteric fractures. This anatomical classification is clinically relevant because intracapsular fractures may compromise femoral head perfusion, increasing the risk of nonunion and avascular necrosis, and thereby influencing the choice of surgical management. Patients who underwent total hip arthroplasty were also excluded, as were those who underwent revision surgery or any other hip fracture-related complications. The final analyses were performed on the 164 patients with intracapsular femoral neck fractures treated with hemiarthroplasty and patients with extracapsular intertrochanteric fractures treated with intramedullary nailing. All patients were mobilized with partial weight-bearing under the supervision of a physiotherapist on postoperative day one. Demographic data, chronic conditions, and other study data, such as laboratory findings, were retrospectively recorded in the patient’s file. The 6-month survival status was examined through the Turkish death registry system.

### 2.2. Risk Models

The American Society of Anesthesiologists (ASA) Physical Status Classification System is a widely used system for assessing a patient’s preoperative health status. It evaluates a patient’s physical condition before surgery, helping anesthesiologists and surgeons assess the risk associated with anesthesia and surgery. The ASA score does not predict specific surgical outcomes but provides a general guideline for anesthesia risk based on the patient’s overall health, which is critical for planning anesthesia and surgery and assessing perioperative risks [22]. The ASA score is a 6-point scale; ASA I denotes a normal, healthy patient, and ASA VI denotes patients who are declared brain-dead [9].

The Hip Fracture Estimator of Mortality Amsterdam (HEMA) is a specific mortality risk assessment tool designed to predict the risk of mortality in patients who have experienced a hip fracture. The HEMA score was developed by the Amsterdam University Medical Center, using a combination of clinical factors including age, in-hospital fracture, signs of malnutrition, a history of myocardial infarction, congestive heart failure, renal failure, malignancy, current pneumonia, and serum urea level. Between 0.5 and 2 points are scored for each variable. Patients can be classified into low-, intermediate-, and high-risk groups based on cumulative scores. The higher the total score, the greater the risk of mortality [10].

The National Hip Fracture Score (NHFS) is another widely used risk-stratification tool designed specifically for patients with hip fractures, based on their preoperative clinical characteristics [11,12]. The NHFS comprises seven variables and estimates the risk of mortality after hip fracture surgery, using age, sex, serum hemoglobin, Abbreviated Mental Test Score (AMT) [23], whether the patient lives in an institution, the number of comorbidities, and a history of malignancy. Between 1 and 4 points are attributed to each variable, with a maximum score of 10. The higher the total score, the greater the risk of mortality. Since this study was retrospective, AMT could not be performed; the presence of impaired cognition was evaluated based on a positive AMT score. NHFS could not be calculated in its original form because AMT scores were not routinely available; therefore, a modified version (mNHFS, excluding AMT) was used.

### 2.3. Frailty Assessment

The frailty status of participants was assessed using the CFS, which is a widely used, semi-quantitative tool that assesses frailty by evaluating an individual’s overall functional and physical health across multiple domains [18]. It is based on the cumulative deficit model, which considers factors such as physical performance, cognitive function, and the presence of chronic diseases or disabilities. The scale provides a global rating of frailty on a 1 to 9 scale, with each score corresponding to a distinct level of frailty, ranging from “very fit” (score 1) to “terminally ill” (score 9). The CFS is designed to be simple, quick to administer, and based on clinical judgment, which makes it particularly useful in routine clinical practice and research settings. Individuals with a score of 4 or higher are considered frail, with higher scores indicating greater severity. The scale integrates factors such as mobility, self-care abilities, cognitive status, and overall energy levels, making it a comprehensive assessment of a person’s ability to live independently and manage daily tasks.

The use of CFS retrospectively was validated in patients with hip fractures in previous studies [24,25]. In our study, it was derived retrospectively from electronic medical records, following the chart-based approach validated in hip fracture populations by Kay et al. [25]. CFS scoring was intended to reflect baseline or pre-fracture frailty, like the patient’s habitual functional status prior to the index hip fracture and before surgery, rather than the acute peri-fracture or postoperative state. A single clinician with formal training in CFS scoring assigned all scores and remained blinded to the 6-month survival status. CFS categories were reconstructed using routinely documented pre-fracture information, including pre-fracture mobility and independent ambulation, use of walking aids, dependence in basic and instrumental activities of daily living, living situation and care requirements, baseline cognitive status, clinician/nursing descriptions of functional reserve and comorbidity-related disability, and prescribed medications. Primary data sources were emergency department admission notes, orthopedic and anesthesia preoperative evaluations, nursing intake documentation, and relevant prior outpatient/inpatient records describing pre-fracture function. Intra-observer reliability was assessed by repeat scoring of 10% of the cohort (*n* = 16) after a 2-week interval, yielding a weighted Cohen’s kappa of 0.86, indicating excellent agreement. Inter-observer reliability could not be evaluated because only one investigator with formal CFS training performed the assessments.

### 2.4. Statistical Analysis

The statistical analysis was performed by the IBM SPSS program version 26.0. The numerical data was examined for its normal distribution using visual and analytical methods. The categorical data was presented as numbers and percentages. The numerical data were presented as mean ± standard deviation (SD) or median [minimum-maximum], according to their distribution. The Chi-square test (χ^2^) was used to assess associations between two categorical variables. Student’s *t*-test was used to compare the means of two independent groups, where the variables were normally distributed. The Mann–Whitney U test (a non-parametric test) was employed when the assumption of normality was violated, or when comparing two independent groups with ordinal or non-normally distributed continuous data. Cox proportional hazard regression analysis was performed to identify independent predictors of six-month mortality. Variables with clinical relevance or a univariate association with mortality (*p* < 0.05) were included in the multivariate analysis. The results were expressed as hazard ratios (HRs) with 95% confidence intervals (CIs). Receiver Operating Characteristic (ROC) curve analysis was conducted to evaluate the diagnostic performance of a continuous predictor variable in discriminating between survivors and non-survivors. The area under the ROC curve (AUC) was calculated to assess test accuracy, with a 95% confidence interval (CI). An AUC value of 0.5 indicates no discriminative ability, while a value of 1.0 indicates perfect discrimination. A *p*-value of less than 0.05 was considered significant for all statistical analyses.

## 3. Results

**Figure 1:**
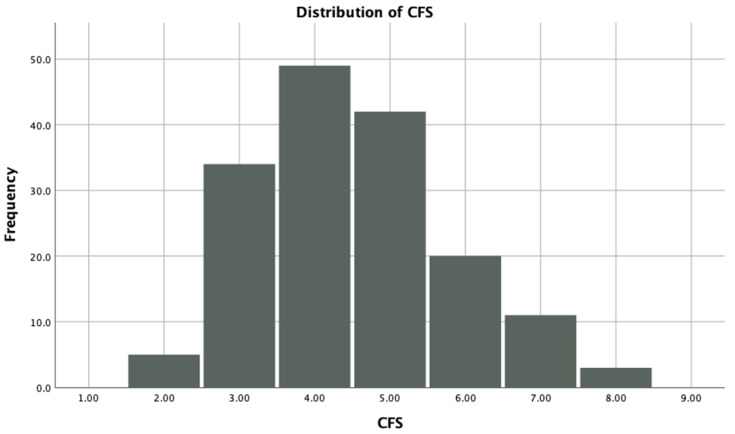
Distribution of CFS categories among the study population.

**Figure 2:**
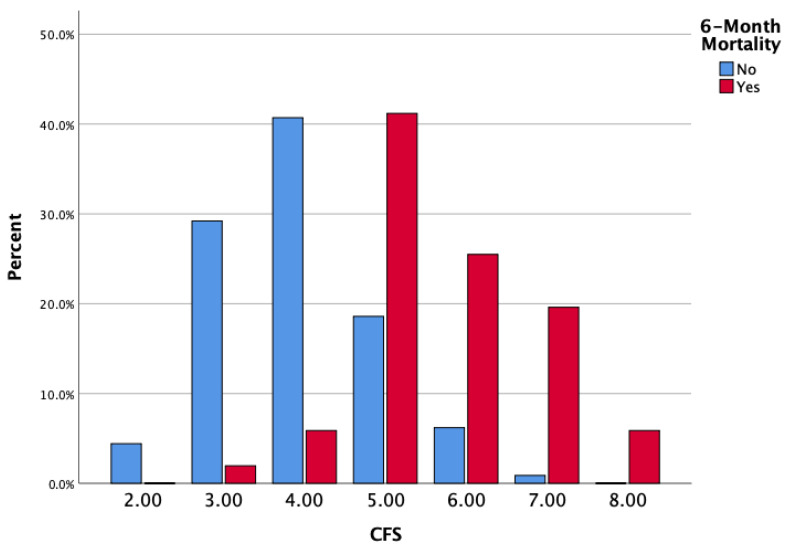
Six-month mortality rates according to frailty levels.

The study population comprised 164 older patients with hip fractures; the mean age was 78.2 ± 8.9, and 64.6% (*n* = 106) of the study group were female. The distribution of baseline frailty status of the entire study group according to the CFS is shown in Figure 1. Frail patients were more prevalent in the ≥85-year subgroup than in the <85-year subgroup (89.5% vs. 72.2%, respectively; *p* = 0.026). Furthermore, 31.1% (*n* = 51) of them were deceased during the 6-month follow-up period. The mortality rates by frailty status are shown in Figure 2.

The age of non-survivors was significantly higher than that of survivors (*p* < 0.001). No differences were found regarding sex and fracture type. The chronic renal disease ratio was significantly higher in the non-survivor group than the survivor group, as well as multimorbidity (*p* = 0.012 and *p* < 0.001, respectively). Dementia and malnutrition rates were significantly higher in the non-survivor group than the survivor group (54.9% vs. 14.2% and *p* < 0.001 for dementia and 96.1% vs. 76.1% and *p* = 0.002 for malnutrition). Percentages of patients living with frailty were found to be 98.0% in the non-survivor group and 66.4% in the survivor group (*p* < 0.001). The median hospitalization time was 8.0 days in the non-survivor group and 5.5 days in the survivor group (*p* = 0.011). The main demographic and characteristic features are shown in Table 1.

The main risk model scores are summarized in Table 2. ASA, HEMA, and mNHFS were significantly higher in the non-survivor group than the survivor group (*p* < 0.001 for all). The median CFS was statistically higher in the non-survivor group than in the survivor group (6.0 [3.0–8.0] vs. 4.0 [2.0–7.0]; *p* < 0.001).

In multivariable Cox regression analysis (Model 1), frailty assessed by CFS was independently associated with 6-month mortality after hip fracture surgery (HR: 4.561, 95% CI: 2.639–7.884; *p* < 0.001). Length of hospital stay was also associated with mortality (HR: 1.049, 95% CI: 1.004–1.095; *p* = 0.033), whereas age (HR: 1.024, 95% CI: 0.963–1.088; *p* = 0.46), female sex (HR:1.067, 95% CI:0.409–2.784; *p* = 0.90), multimorbidity (HR:1.407, 95% CI: 0.553–3.585; *p* = 0.47), and extracapsular fracture type (HR:0.519, 95% CI:0.192–1.400; *p* = 0.20) were not significant predictors. In Model 2 using a categorical frailty definition, frailty remained significantly associated with 6-month mortality (HR: 13.887; 95% CI 1.712–112.622; *p* = 0.014). Sex, multimorbidity, and extracapsular fracture type were not significantly associated with mortality (Table 3). Subgroup analysis by age group is shown in the Supplementary Table S1.

**Figure 3:**
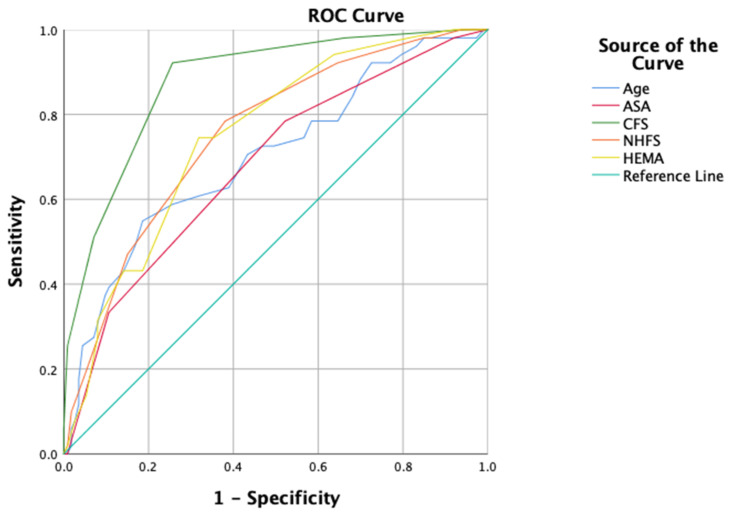
ROC curve of the risk models.

The predictive ability of risk models for 6-month mortality and frailty evaluation is assessed using ROC analysis in Table 4. All parameters were predictive of 6-month mortality in hip fracture patients, with the highest AUC of 0.879 (95% CI: 0.824–0.934) in CFS (*p* < 0.001). Pairwise comparisons of correlated ROC curves showed that CFS had a significantly higher AUC than ASA, age, HEMA, and mNHFS (all *p*-values ≤ 0.001 by the DeLong test). Figure 3 shows the ROC curves.

## 4. Discussion

Hip fractures represent a significant health issue in older adults, characterized by high mortality rates. The prediction of mortality risk is crucial for guiding clinical decision-making, optimizing treatment strategies, and personalizing interventions. In the present study, frailty, as assessed by the CFS, was identified as an independent risk factor for mortality, irrespective of age, sex, comorbidity burden, and hospital length of stay. Notably, the CFS demonstrated superior predictive accuracy for six-month mortality compared to traditional perioperative risk models. These findings emphasize the importance of a comprehensive frailty assessment in hip fracture patients to improve risk stratification and ultimately enhance clinical outcomes.

Frailty has emerged as a critical factor in understanding the variability of clinical outcomes and mortality in older adults following hip fractures, yet its role remains controversial. The study assessed frailty in patients aged 65 years and older with hip fractures in Australia between 2014 and 2021 using the Hospital Frailty Risk Score and found that higher frailty risk was associated with increased risk of refracture and mortality [26]. Meanwhile, Mitchell et al. revealed that frailty was associated with readmission to the hospital but not mortality in institutionalized older patients with hip fractures [27]. In our study, frailty assessed using the CFS was identified as an independent predictor of six-month mortality. These findings underscore the importance of frailty assessment in hip fracture patients and suggest that standardized tools such as the CFS can provide valuable insights into mortality risk and guide individualized patient care.

In the literature, some studies compare the different risk prediction models of mortality. In 2015, Karres et al. evaluated six risk models in a retrospective study, including the Charlson comorbidity index and models specific to hip fracture patients. They revealed that NHFS showed the most promise among the proposed models, with good discriminative performance; however, none of these models was perfect [28]. A cohort study by Ikram et al. found that the predictive ability of CFS and NHFS was similar in 30-day mortality [29]. Another retrospective study reported that CFS demonstrated superior discriminative ability for predicting mortality after proximal femur fractures compared with ASA and chronological age [30]. Additionally, in our study, CFS shows a reasonable predictive ability for 6-month mortality compared with ASA, mNHFS, and chronological age. A likely explanation is that CFS reflects baseline functional status and cognitive vulnerability, which are strong determinants of recovery capacity after a major stressor such as hip fracture surgery. Traditional perioperative models, such as ASA and NHFS, primarily capture the comorbidity burden and perioperative physiological risk but may not fully assess pre-fracture dependency, mobility, cognition, or the ability to withstand postoperative complications, including delirium, immobility, malnutrition, and deconditioning.

Unlike some traditional perioperative risk models that primarily focus on physiological parameters or comorbidities, frailty incorporates a holistic point of view of a patient’s functional and cognitive status. Additionally, integrating frailty into existing risk prediction models could enhance their predictive accuracy. Therefore, the Orthopedic Frailty Score (OFS) has been introduced as a tool to assess frailty, aiming to predict short-term postoperative mortality in patients with hip fractures, evaluating the five factors, including congestive heart failure, institutionalization, non-independent functional status, age ≥ 85, and a history of malignancy [31]. Moreover, another frailty index was developed from the Irish Hip Fracture Database; this 21-item frailty index was a significant predictor of outcomes and added value to traditional risk markers [32]. Similar to these frailty indices, in CFS, independence and disease burden were evaluated based on the clinician’s overall judgment. This broader perspective may explain why frailty performs well as an independent predictor of mortality.

We have acknowledged that this study had some limitations. First of all, this is a retrospective study, and the retrospective use of CFS in patients with hip fractures could cause some misinterpretations. Furthermore, because AMT is a key component of NHFS, using a modified NHFS without AMT likely underestimates the original tool’s true predictive ability. This limitation may partly explain the lower AUC observed for NHFS in our study and should be considered when interpreting comparative model performance. Secondly, only the 6-month mortality was evaluated; short and long-term mortality data were missing. However, evaluating 6-month mortality provides meaningful insights into the critical recovery and rehabilitation period for hip fracture patients, an important benchmark for clinicians. The current study focused solely on mortality, neglecting other important patient-related outcomes, such as functional recovery, quality of life, or readmission rates, which are also critical in older adults with hip fractures. Due to the study’s retrospective design, patient-related psychological and social factors were not recorded. Furthermore, the chronic disease burden was considered; specific comorbid conditions and their severity were not analyzed individually, which may have limited insights into their interactions with frailty. On the other hand, our findings suggest that CFS provides clinically notable prognostic information beyond traditional perioperative risk models. Given its simplicity and strong association with 6-month mortality, routine CFS assessment at admission could be integrated into orthogeriatric pathways as an early triage tool alongside ASA/NHFS. From an operational standpoint, higher CFS can justify earlier geriatric co-management, more frequent nursing assessments, and discharge planning that anticipates the need for inpatient rehabilitation, home health services, or caregiver support.

The use of a simple, validated scale, such as CFS, makes the findings applicable across a wide range of healthcare systems, particularly in resource-limited settings where more complex risk models may not be feasible, and frailty assessment would be a complementary alternative. With proper education and training, the CFS can be easily performed by non-geriatricians and may help mitigate age-related bias in surgical decision-making, enabling more objective and equitable assessment of patient risk. Frailty should integrate, not replace, validated perioperative risk tools and should be used to support medium-term prognostication and care planning, like postoperative monitoring, orthogeriatric co-management, rehabilitation, and discharge needs, not to delay or deny surgery or analgesia. Further studies investigating the long-term predictive ability of the CFS and its integration with other models could be planned to evaluate the validity of our results.

## Figures and Tables

**Table 1:** The baseline characteristics of the patients with hip fracture according to 6-month survival status.

	Survivor (*n* = 113)	Non-Survivor (*n* = 51)	*p*
Age, years	76.2 ± 8.5	82.4 ± 8.5	<0.001
Sex, female	76 (67.3)	30 (58.8)	0.30
Fracture Type			
Intracapsular	65 (57.5)	31 (60.8)	0.70
Extracapsular	48 (42.5)	20 (39.2)	
Chronic Renal Disease	18 (15.9)	17 (33.3)	0.012
Chronic Heart Failure	6 (5.3)	5 (9.8)	0.32
Malignancy	13 (11.5)	6 (11.8)	0.96
Multimorbidity	44 (38.9)	35 (68.6)	<0.001
Malnutrition	86 (76.1)	49 (96.1)	0.002
Dementia	16 (14.2)	28 (54.9)	<0.001
Osteoporosis	16 (14.2)	7 (13.7)	0.15
Frailty, CFS	75 (66.4)	50 (98.0)	<0.001
Length of hospital stay, days	5.5 [1.0–69.0]	8.0 [1.0–182.0]	0.011
Vitamin D level	11.7 [1.9–67.4]	12.8 [3.0–51.2]	0.68

The variables are presented as number (%), mean ± SD, or median [minimum–maximum].

**Table 2:** Assessment of the risk models of the patients with hip fracture according to 6-month survival status.

	Survivor (*n* = 113)	Non-Survivor (*n* = 51)	*p*
ASA	3.0 [1.0–5.0]	3.0 [1.0–4.0]	<0.001
HEMA	3.0 [0.0–7.5]	4.0 [1.0–7.5]	<0.001
mNHFS	4.0 [0.0–9.0]	5.0 [1.0–9.0]	<0.001
CFS	4.0 [2.0–7.0]	6.0 [3.0–8.0]	<0.001

ASA; American Society of Anesthesiologists, HEMA; the Hip fracture Estimator of Mortality of Amsterdam, mNHFS; the Nottingham Hip Fracture Scale (NHFS without AMT), CFS; Clinical Frailty Scale. The variables were presented as median [minimum-maximum].

**Table 3:** The Cox regression analysis of the factors related to 6-month mortality.

	Hazard Ratio	95% Confidence Interval	*p*
Model 1			
Age, years	1.024	0.963–1.088	0.46
Sex, female	1.067	0.409–2.784	0.90
Multimorbidity	1.407	0.553–3.585	0.47
Fracture type, extracapsular	0.519	0.192–1.400	0.20
Length of hospital stay, days	1.049	1.004–1.095	0.033
Frailty, CFS	4.561	2.639–7.884	<0.001
Model 2			
Age, years	1.078	1.027–1.132	0.002
Sex, female	1.090	0.487–2.438	0.84
Multimorbidity	2.059	0.928–4.568	0.076
Fracture type, extracapsular	0.686	0.308–1.530	0.36
Length of hospital stay, days	1.044	0.997–1.094	0.066
Frailty (CFS > 4)	13.887	1.712–112.622	0.014

CFS; Clinical Frailty Scale.

**Table 4:** The predictive ability of the risk models according to ROC analysis.

	AUC	95% CI	*p*	Delta AUC (95% CI)	*p*
CFS	0.879	0.824–0.934	<0.001	-	-
Age	0.700	0.611–0.789	<0.001	−0.179 (−0.269–−0.089)	<0.001
ASA	0.679	0.590–0.767	<0.001	−0.200 (−0.294–−0.105)	<0.001
HEMA	0.750	0.673–0.826	<0.001	0.129 (0.050–0.208)	0.001
mNHFS	0.752	0.674–0.830	<0.001	0.127 (0.055–0.198)	0.001

AUC, Area Under Curve; CI, Confidence Interval, ASA; American Society of Anesthesiologists, HEMA; the Hip fracture Estimator of Mortality of Amsterdam, mNHFS; the Nottingham Hip Fracture Scale (NHFS without AMT), CFS; Clinical Frailty Scale.

## Data Availability

Data are available on reasonable request from the corresponding author, Merve Güner, at mguner54@gmail.com.

## Supplementary Materials

The following supporting information can be downloaded at https://www.mdpi.com/article/10.3390/jcm15145625/s1, Table S1: Subgroup analysis by age (≥85 years) of Multivariable Cox regression for 6-month mortality after hip fracture surgery.
