# Supplementary material for: Comparing Frailty and Traditional Risk Models in Predicting 6-Month Mortality After Hip Fracture in Older Adults: A Retrospective Study from a Single Center
Source: J Clin Med. 2026 Jul 17;15(14):5625. doi: 10.3390/jcm15145625 (PMC13412865; doi:10.3390/jcm15145625)
Supplement: Supplementary file 1 [file jcm-15-05625-s001.zip › jcm-4170422-supplementary.pdf]

Table S1: Subgroup analysis by age (≥85 years) of Multivariable Cox regression for 6-month mor-tality after hip fracture surgery

Model 1 (Age<85 years)	Hazard Ratio	95% Confidence Interval	p
**Length of hospital stay, days**	1.045	0.995–1.098	0.081
**Fracture type, extracapsular**	0.620	0.199–1.932	0.410
**Multimorbidity**	2.082	0.676–6.410	0.201
**Sex, female**	1.430	0.449–4.550	0.545
**Frailty, CFS**	**4.254**	**2.267–7.982**	**<0.001**
**Model 2 (Age ≥ 85 years)**			
**Length of hospital stay, days**	1.106	0.931–1.314	0.253
**Fracture type, extracapsular**	0.318	0.041–2.443	0.271
**Multimorbidity**	0.419	0.045–3.864	0.443
**Sex, female**	0.580	0.084–4.008	0.581
**Frailty, CFS**	**5.964**	**1.685–21.103**	**0.006**
